# Social Distancing, Mask Use, and Transmission of Severe Acute Respiratory Syndrome Coronavirus 2, Brazil, April–June 2020

**DOI:** 10.3201/eid2708.204757

**Published:** 2021-08

**Authors:** Marcelo Rodrigues Gonçalves, Rodrigo Citton Padilha dos Reis, Rodrigo Pedroso Tólio, Lucia Campos Pellanda, Maria Inês Schmidt, Natan Katz, Sotero Serrate Mengue, Pedro C. Hallal, Bernardo L. Horta, Mariangela Freitas Silveira, Roberto Nunes Umpierre, Cynthia Goulart Bastos-Molina, Rodolfo Souza da Silva, Bruce B. Duncan

**Affiliations:** Universidade Federal do Rio Grande do Sul, Porto Alegre, Brazil (M.R. Gonçalves, R.C.P. Reis, R.P. Tólio, M.I. Schmidt, N. Katz, S.S. Mengue, R.N. Umpierre, C.G. Bastos-Molina, R.S. da Silva, B.B. Duncan);; Hospital de Clínicas de Porto Alegre, Porto Alegre (M.R. Gonçalves, M.I. Schmidt, R.N. Umpierre, C.G. Bastos-Molina, R.S. da Silva, B.B. Duncan);; Universidade Federal de Ciências da Saúde de Porto Alegre, Porto Alegre (L.C. Pellanda);; Municipal Health Department, Porto Alegre (N. Katz);; Universidade Federal de Pelotas, Pelotas, Brazil (P.C. Hallal, B.L. Horta, M.F. Silveira);; Universidade do Vale dos Sinos (UNISINOS), São Leopoldo, Brazil (C.G. Bastos-Molina)

**Keywords:** communicable disease control, masks, pandemics, social distance, social isolation, COVID-19, coronavirus disease, SARS-CoV-2, severe acute respiratory syndrome coronavirus 2, viruses, respiratory infections, zoonoses

## Abstract

We assessed the associations of social distancing and mask use with symptomatic, laboratory-confirmed severe acute respiratory syndrome coronavirus 2 infection in Porto Alegre, Brazil. We conducted a population-based case-control study during April–June 2020. Municipal authorities furnished case-patients, and controls were taken from representative household surveys. In adjusted logistic regression analyses of 271 case-patients and 1,396 controls, those reporting moderate to greatest adherence to social distancing had 59% (odds ratio [OR] 0.41, 95% CI 0.24–0.70) to 75% (OR 0.25, 95% CI 0.15–0.42) lower odds of infection. Lesser out-of-household exposure (vs. going out every day all day) reduced odds from 52% (OR 0.48, 95% CI 0.29–0.77) to 75% (OR 0.25, 95% CI 0.18–0.36). Mask use reduced odds of infection by 87% (OR 0.13, 95% CI 0.04–0.36). In conclusion, social distancing and mask use while outside the house provided major protection against symptomatic infection.

The coronavirus disease (COVID-19) pandemic has spread worldwide (*1*). The rapid transmission of the causative agent, severe acute respiratory syndrome coronavirus 2 (SARS-CoV-2), has produced a high death toll, threatening health systems and creating huge challenges for governments and societies.

Until advances in the development and distribution of vaccines or treatment reduce the risk for COVID-19 complications to levels permitting near-normal day-to-day functioning, societies continue to require simple public health approaches to control pandemic spread, including mask use and social distancing. Several cohort studies in hospital settings have shown benefits of both interventions (*2*). However, in community settings, where these approaches have the greatest potential to limit viral spread and halt the pandemic, documented support for their use comes mostly from ecologic studies and, indirectly, from findings related to previous pandemics of other coronaviruses. Only a few studies (*3*), including a retrospective case-control study of asymptomatic contacts (*4*), a randomized trial (*5*), and a study at sea (*6*), have evaluated their effectiveness against community transmission of SARS-CoV-2 on the basis of individual-level exposure and outcome measurements. Their relevance remains embroiled in controversy. To help close this gap, we evaluated the association of mask use and social distancing with incident, symptomatic, laboratory-confirmed SARS-CoV-2 infection in a population-based case-control study.

## Methods

We conducted a population-based, case-control study in Porto Alegre, the capital of Rio Grande do Sul State, Brazil, which has an estimated population of 1,483,771 (*7*). The ethics committee of the Hospital de Clínicas de Porto Alegre approved our study (approval no. 31499420.5.0000.5327), and the Brazilian National Ethics Committee (approval no. 30415520.2.0000.5313) approved the accompanying seroprevalence surveys. All participants gave prior informed consent, in written form by the controls and verbally for case-patients.

On March 19, 2020, state officials mandated school and nonessential business closure and travel restrictions and ordered citizens to stay at home unless going to essential services (*8*). On May 8, 2020, Porto Alegre’s mayor issued a series of orders and recommendations for mask use. These mandates, with only slight alterations, remained in force in Porto Alegre throughout the period of this study. However, social distancing and mask use were not universally adopted; prominent leaders questioned their necessity and supported widely publicized gatherings, frequently without mask use.

We obtained case-patients from the Municipal Health Department, given that notification of COVID-19 cases is mandatory. The list consisted of all persons (excluding healthcare professionals) who tested positive for SARS-CoV-2 by reverse transcription PCR or antibody testing through June 19, 2020, in Porto Alegre. With rare exceptions, case-patients were receiving medical care, because testing during this period was limited and available only for symptomatic persons. Cases were identified in hospitals and primary care settings. We then contacted persons >18 years of age whose date of symptom onset was on or after April 28, 2020. Before deeming a case-patient nonrespondent, we attempted >10 calls on different days at different hours, as well as attempting contact through short message service, WhatsApp, other social media, and physical mail. We excluded persons working in healthcare settings because our focus was community transmission. We also excluded deceased persons and persons who resided outside the municipality. When the case-patient could not be interviewed, we obtained responses from a proxy (i.e., a close contact, either a family member or caretaker).

Controls were the seronegative persons in 3 representative community surveys of SARS-CoV-2 antibody prevalence in Porto Alegre conducted during May 9–11, May 23–25, and June 26–28, 2020 (*9*,*10*). For the surveys, 50 of Porto Alegre’s census tracts were selected with probability proportional to size. Within each, during each survey, 10 households were selected systematically; if no one was home or residents refused participation, we used the neighboring residence. A resident of each home was then selected at random for interview.

Controls underwent a brief interview, including questions on social distancing, mask use, and sociodemographics. Seropositivity was determined by a point-of-care rapid antibody test (L.C. Pellanda et al., unpub. data, https://www.medrxiv.org/content/10.1101/2020.05.06.20093476v1). For case-patients, we conducted telephone interviews by using the same questions applied in the surveys.

Trained interviewers queried case-patients and controls using standardized questionnaires: “Regarding the social distancing recommended by health authorities, that is, staying at home and avoiding contact with other people, how much do you think you have managed to do?” Reply choices were 1, very little; 2, little; 3, some; 4, a great deal; and 5, practically isolated from everyone.

In response to “What has been your routine of activities?” participants opted among the following choices: 1, go out every day, all day, to work or other regular activity; 2, go out every day for some activity; 3, go out from time to time to shop and stretch my legs; 4, go out only for essential things like buying food; and 5, stay at home all the time. We created and categorized a social distancing score by summing responses to each of these questions when taken as an ordinal scale.

All case-patients were asked about mask use, but controls were asked about mask use only during the last seroprevalence survey. In response to “Do you use a mask when you leave home?” case-patients opted between yes and no and controls among yes, sometimes, and no. For modeling, we merged the replies yes and sometimes. For case-patients and controls, we defined income as mean head-of-household monthly income on the respondent’s census tract.

We calculated sample size by using an α of 0.05 and 80% power: to detect an odds ratio of 2 would require 93 case-patients and 372 controls. We described continuous variables by mean (SD) or median (interquartile range) and categorical variables by frequency (percentage). When information on household size was missing, we used the mean household size of the respondent’s census tract. Participants with other missing values were excluded from analyses. We investigated associations of social distancing and mask use through prespecified logistic regression analyses. We defined the pandemic moment as the date of symptom onset for case-patients and as 10 days before the date of interview for controls. We performed all analyses by using the statistical software package R version 4.0.2 (*11*).

## Results

**Figure 1:**
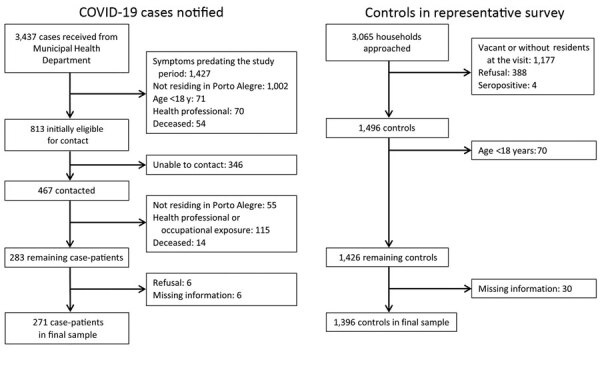
Flowchart of COVID-19 case-patient and controls, Porto Alegre, Brazil, April–June 2020. COVID-19, coronavirus disease.

Of all initial case-patients, after excluding deceased persons and those who were not part of the target population, 813 case-patients were eligible for contact (Figure 1). We established contact with 467 (57.4%) and found an additional 184 ineligible. Of the remaining 283 persons, 12 refused participation or provided incomplete social distancing information, leaving 271 case-patients. We interviewed 237 (87.5%) directly and 34 (12.5%) by proxy. If the proportion of actual eligible case-patients among the 813 initially eligible persons was the same as among those contacted (283/467), the 271 cases represent a response rate of 55.0% among those actually eligible. Comparison of the Municipal Health Department case data showed that case-patients in the final sample differed little from those not included in terms of sex (43.9% [95% CI 37.9%–50.0%] men among those included vs. 48.3% [95% CI 44.7%–58.6%] men among those excluded) and age (46.0 [95% CI 44.0–48.0] years for those included vs. 48.0 [95% CI 46.2–49.8] years for those excluded).

For controls, of 3,065 households approached, 1,177 (38.4%) were vacant or without residents at home, residents refused in 388 (12.7%) households, and 4 seropositive persons were excluded; a total of 1,496 (48.8%) potential controls were contacted (*12*). An additional 70 were <18 years old and data on race were missing for 30, leaving 1,396 (45.5%) for analyses. Comparison of controls in the final sample with the 30 persons for whom data were missing demonstrated they were also similar in sex (38.5% [95% CI 35.9%–41.0%] men for final controls vs. 36.7% [95% CI 21.9%–54.5%] men for those with data missing) and age (49.7 [95% CI 48.8–50.6] for final controls vs. 52.3 [95% CI 44.1–60.5] years for those with data missing).

Our controls were more frequently women and were somewhat older than the average of the adult population of Porto Alegre (Table 1) (*13*). Case-patients, compared to controls, were more frequently men, Black, and younger; had a lower level of education; and lived in larger households (Table 2). Case-patients were less likely to adhere to social distancing. In the variable summarizing social distancing, case-patients more frequently practiced least (16.2% vs. 7.2% for controls) or little (26.6% vs. 15.5% for controls) social distancing. Mask use was commonly reported. After we excluded those reporting staying at home all the time, only 5 (1.2%) controls and 14 (7.1%) case-patients reported not using masks when out.

**Table 1:** Comparison of sociodemographic characteristics between control subjects and the population of Porto Alegre, Brazil, in study of face masks, social distancing, and transmission of severe acute respiratory syndrome coronavirus 2, April–June 2020

Characteristic	Porto Alegre, %	Controls, %
Sex		
M	47.8	38.5
F	52.2	61.5
Age group, y		
18–29	20.2	15.5
30–39	18.4	17.9
40–49	15.8	16.1
50–59	18.8	17.6
>60	26.8	33.0
Race		
White	75.3	75.4
Nonwhite	24.7	24.6

**Table 2:** Sociodemographic and social distancing data of case-patients and controls, Porto Alegre, Brazil, April–June 2020*

Characteristic	Case-patients, n = 271	Controls, n = 1,396
Sex		
M	119 (43.9)	537 (38.5)
F	152 (56.1)	859 (61.5)
Mean age, y, ±SD	46.0 ±17.2	49.7 ±17.5
Education		
University	98 (36.2)	722 (51.7)
High school complete	88 (32.5)	388 (27.8)
High school incomplete	85 (31.4)	286 (20.5)
Race		
White	197 (72.7)	1053 (75.4)
Mixed race	35 (12.9)	182 (13.0)
Black	36 (13.3)	149 (10.7)
Other	3 (1.1)	12 (0.9)
Household size ±SD	2.9 ±1.2†	2.5 ±1.4
Monthly income, Brazilian real, head of household‡	1,575 (IQR 965–3,365)	2,205 (IQR 1,089–3,390)
Epidemiologic week ±SD	21.9 ±1.6	21.0 ±2.9
Adherence to social distancing		
Very little	32 (11.8)	56 (4.0)
Little	32 (11.8)	81 (5.8)
Moderate—some	43 (15.9)	260 (18.6)
High—a great deal	88 (32.5)	651 (46.6)
Practically isolated from everybody	76 (28.0)	348 (24.9)
Daily routine		
Go out every day, all day, to work or other regular activity	118 (43.5)	251 (18.0)
Go out every day for some activity	12 (4.4)	102 (7.3)
Go out from time to time to shop and stretch my legs	30 (11.1)	192 (13.8)
Go out only for essential things like buying food	74 (27.3)	696 (49.9)
Stay at home all the time	37 (13.7)	155 (11.1)
Social distancing score ±SD	6.2 ±2.5	7.1 ±2.0
Social distancing		
Least	44 (16.2)	100 (7.2)
Little	72 (26.6)	216 (15.5)
Much	98 (36.2)	729 (52.2)
Most	57 (21.0)	351 (25.1)
Mask use§		
No	14 (7.1)	5 (1.2)
Sometimes	NA	10 (2.4)
Always	184 (92.9)	405 (96.4)

*Values are no. (%) except as indicated. IQR, interquartile range; NA, not applicable. †Excluding 4 case-patients living in nursing homes. ‡Median of mean head-of-household income of respondent´s census tract. §Including only cases with pandemic moment equivalent to that of the last seroprevalence survey and excluding case-patients and controls reporting to stay at home all the time.

**Figure 2:**
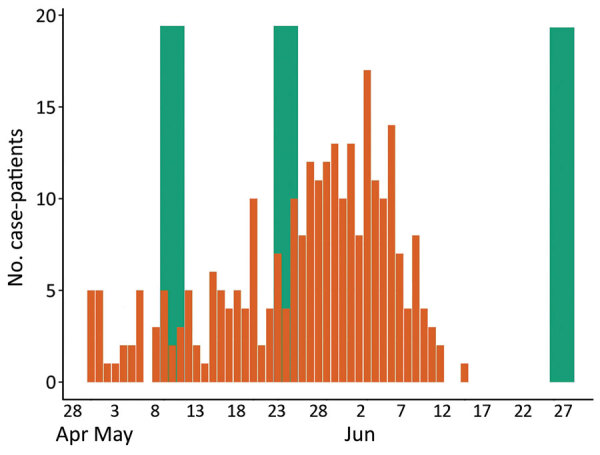
Number of case-patients (n = 271) by date of coronavirus disease symptom onset in study of face masks, social distancing, and transmission of severe acute respiratory syndrome coronavirus 2, Porto Alegre, Brazil, April–June 2020. Green bars indicate dates of interviews of controls (n = 1,396): May 9–11, May 23–25, and June 26–28.

We compared the temporal distribution of symptom onset of case-patients and the 3 interview periods for controls (Figure 2). On average, symptom onset in case-patients was slightly less than a week before the interview date of controls (Table 2).

In crude analyses (Table 3, model 1), moderate or high adherence to social distancing and being practically isolated from everyone all reduced risk for infection. Multiple adjustments (models 2 and 3) produced little change. In model 3, those with moderate adherence to social distancing were 72% (OR 0.28, 95% CI 0.16–0.49) and those with high adherence 75% (OR 0.25, 95% CI 0.15–0.42) less likely to become infected. Persons who reported they were practically isolated from everyone were 59% (OR 0.41, 95% CI 0.24–0.70) less likely to become infected. When we excluded proxy interviews (model 4), the association of being practically isolated from everyone became stronger (OR 0.34, 95% CI 0.20–0.60).

**Table 3:** Association of social distancing with severe acute respiratory syndrome coronavirus 2 infection, Porto Alegre, Brazil, April–June 2020

Characteristic, n = 1,667	Odds ratio (95% CI)			
	Model 1*	Model 2†	Model 3‡	Model 4§
Social distancing: How much have you managed to do?¶				
Little	0.69 (0.38–1.26)	0.72 (0.39–1.32)	0.65 (0.35–1.21)	0.65 (0.34–1.22)
Moderate (Some)	0.29 (0.17–0.50)	0.30 (0.17–0.52)	0.28 (0.16–0.49)	0.30 (0.17–0.53)
High (A great deal)	0.24 (0.15–0.39)	0.28 (0.17–0.47)	0.25 (0.15–0.42)	0.26 (0.16–0.44)
Practically isolated from everyone	0.38 (0.23–0.63)	0.44 (0.26–0.75)	0.41 (0.24–0.70)	0.34 (0.20–0.60)
What has been your routine of activities?#				
Go out every day for some activity	0.25 (0.13–0.46)	0.27 (0.14–0.50)	0.26 (0.13–0.49)	0.25 (0.12–0.48)
Go out from time to time for some activity	0.33 (0.21–0.51)	0.38 (0.24–0.59)	0.39 (0.24–0.61)	0.38 (0.23–0.60)
Go out just for essential activities	0.23 (0.16–0.31)	0.24 (0.17–0.34)	0.25 (0.18–0.36)	0.25 (0.18–0.35)
Stay at home all the time	0.51 (0.33–0.77)	0.51 (0.31–0.80)	0.48 (0.29–0.77)	0.25 (0.13–0.44)
Social distancing summary classification**				
Little	0.76 (0.49–1.19)	0.80 (0.51–1.26)	0.73 (0.46–1.16)	0.75 (0.47–1.20)
Much	0.31 (0.20–0.46)	0.35 (0.23–0.54)	0.33 (0.22–0.52)	0.33 (0.22–0.52)
Most	0.37 (0.24–0.58)	0.40 (0.25–0.65)	0.38 (0.23–0.62)	0.27 (0.16–0.46)

*Crude model. †Model 1 with addition of sex, age, educational attainment, race, and income. ‡Model 2 with addition of household size and pandemic moment. §Model 3 but excluding case-patients for whom a proxy provided the interview. ¶Reference category: very little. #Reference category: go out every day, all day, to work or other regular activity. **Reference category: least.

In similar models (Table 3), lesser activity of any degree reduced the odds of infection compared to leaving home daily for the whole day. Relatively little confounding was present, and in models adjusted for all covariates, going out for some activities every day reduced odds by 74% (OR 0.26, 95% CI 0.13–0.49), going out from time to time reduced odds by 61% (OR 0.39, 95% CI 0.24–0.61), and going out just for essential activities reduced odds by 75% (OR 0.25, 95% CI 0.18–0.36). After we excluded proxy interviews (model 4), staying home all the time also provided a major reduction in odds (OR 0.25, 95% CI 0.13–0.44).

When these 2 measures were joined in a categorical summary measure of social distancing (Table 3), practicing much distancing reduced the adjusted odds of becoming infected by 67% (OR 0.33, 95% CI 0.22–0.52) and most distancing reduced odds by 62% (OR 0.38, 95% CI 0.23–0.62), in comparison to least distancing. After excluding proxy interviews, the association became graded; odds were 73% lower (OR 0.27, 95% CI 0.16–0.46) among persons who practiced the most social distancing.

Because information on mask use was only obtained during the third seroprevalence survey, we compared use for the 464 controls in this survey with 229 case-patients of a similar pandemic moment (symptom onset <10 days before the second survey). Considering all those with mask data during this period, crude analyses demonstrated that mask use reduced odds of infection by 88% (OR 0.12, 95% CI 0.04–0.30), and after adjustments, including the summary distancing score (Table 4, model 3), by 90% (OR 0.10, 95% CI 0.03–0.25). No interaction was seen between mask use and social distancing (OR 0.96, 95% CI 0.60–1.58). The association was similar in the restricted sample, which removed those who reported staying home all the time (87%; OR 0.13, 95% CI 0.04–0.36) and, in addition, when proxy respondents were removed (88%; OR 0.12, 95% CI 0.04–0.35). In a sensitivity analysis in which “sometimes” mask use was joined with “no” rather than with “always,” mask use reduced odds of infection (model 3) by 64% (OR 0.36, 95% CI 0.17–0.74).

**Table 4:** Association of mask use with severe acute respiratory syndrome coronavirus 2 infection, Porto Alegre, Brazil, April–June 2020

Sample	Odds ratio (95% CI)			
	Model 1*	Model 2†	Model 3‡	
All, n = 693	0.12 (0.04–0.30)	0.10 (0.03–0.25)	0.10 (0.03–0.25)	
Restricted sample, n = 618§	0.16 (0.05–0.42)	0.12 (0.04–0.35)	0.13 (0.04–0.36)
Restricted sample, proxies removed, n = 609¶	0.16 (0.05–0.44)	0.12 (0.03–0.34)	0.12 (0.04–0.35)

*Crude model. †Model 1 with addition of sex, age, educational attainment, race, income, and household size. ‡Model 2 with addition of social distancing score. §Excluding those who stated that they stay at home all the time. ¶Excluding proxy responses.

Finally, when we adjusted social distancing associations for mask use in an analysis limited to controls from the third survey and cases of similar pandemic moment, we found little change in associations with social distancing. There was 50% (OR 0.51, 95% CI 0.27–0.93) lesser risk for infection with little social distancing, 67% (OR 0.33, 95% CI 0.19–0.60) with much social distancing, and 59% (OR 0.41, 95% CI 0.21–0.80) with the most social distancing.

## Discussion

In this population-based case-control study of COVID-19 conducted during a period of low-level to mid-level viral transmission in a major city in Brazil, mask use and adherence to social distancing resulted in major protection against symptomatic SARS-CoV-2 infection. Even after adjusting for various risk factors, adults who reported moderate or greater adherence to distancing recommendations reduced their odds of infection by one half to two thirds, and those who reported using masks when out reduced their risk by 87%. Because we excluded persons in healthcare settings, our findings directly address the use of these measures for protection against COVID-19 in the general community.

Evidence supporting the use of nonpharmacologic public health measures to slow viral spread of SARS-CoV-2 in communities has come mainly from ecologic studies documenting large inverse associations between greater use of these measures and viral spread (*14*–*17*). Evidence based on individual-level analyses, which come almost exclusively from studies of severe acute respiratory syndrome (SARS) and Middle East respiratory syndrome or from investigations in hospitals, have findings similar to ours: that risk approximately doubled with each additional meter of proximity to known infected persons, and that mask use reduced risk for transmission by 85% (OR 0.15, 95% CI 0.07–0.34) (*2*). Similar, although weaker, protection in a SARS-CoV-2 outbreak in a specific setting (the USS Roosevelt aircraft carrier) was found with greater use of face coverings (OR 0.30, 95% CI 0.17–0.52), avoidance of common areas (OR 0.56, 95% CI 0.37–0.86), and increased distance from others (OR 0.52, 95% CI 0.34–0.79) (*6*).

Very few individual-level studies have been reported on the effect of these measures on community transmission of SARS-CoV-2 (*18*). A case-control study of asymptomatic contacts in Thailand documented a risk reduction of 77% with mask use and 85% with distancing greater than 1 meter (*4*), and a study from Wuhan, China, showed mask use at home during the lockdown provided protection (*19*). An additional report ascertained that greater mask use reduced risk for predicted COVID-19 by 63% (S. Kwon et al., unpub. data, https://www.medrxiv.org/content/10.1101/2020.11.11.20229500v1). Finally, a cross-sectional study from Vermont, USA, with only 10 cases showed some protection in crude analyses (*20*).

A randomized trial in Denmark (*5*) that suggested lower, nonsignificant protection (OR 0.82, 95% CI 0.54–1.23) of mask use, although based on a potentially stronger design, had major methodologic problems (*21*,*22*). First, mask use was limited; only 46% reported full adherence. Second, 84% of outcomes were detected by antibody testing, leading an editorial accompanying the publication (*21*) to note that, given the extremely low incidence of cases, “all of the antibody-positive results in both intervention and control groups could have been false positives.” When the study analyzed the subset of healthcare-diagnosed cases (15 participants), masks provided a greater, though not statistically significant, protection (OR 0.52, 95% CI 0.18–1.53). Third, the trial’s short study periods (1 month), coupled with low antibody test sensitivity in early disease (30% <7 days and 72% during days 8–14) (*23*), could have resulted in the inclusion during the initial 2 weeks of case-patients who had contacted the disease before trial initiation. Similarly, during the final 2 weeks of the study period, some infections could have been missed by antibody testing, also not being detected by home-based reverse transcription PCR of uncertain sensitivity at close-out. Finally, because the intervention did not include face mask use by other household members, some cases could have resulted from home exposure, limiting the applications of the trial to current community settings, in which a greater fraction of other household members would also be using masks when out.

Our study provides estimates for easily interpretable measures—percentage effectiveness of social distancing and masking in protecting against infection—in the general community, the setting of greatest relevance for controlling the pandemic. The study occurred during a period of low to moderate transmission. Rio Grande do Sul State seroprevalence data suggest that ≈0.5% of the population became infected and 57 (3.8/100,000 population) COVID-19 deaths occurred in Porto Alegre during our ≈2-month study period (*24*).

The first potential limitation of our study was that response rates for case-patients (55.0%) and controls (45.5%) were low, and differential nonparticipation could introduce selection bias. Whereas not being available to participate could be associated with less social distancing, additional factors could explain the low response. Among case-patients, the frequent address changes identified when contact was achieved suggest that many case-patients on the initial list were ineligible because they were nonresidents who had furnished a false address to gain access to care. In addition, telemarketing and telephone scams lead many to ignore calls from unknown numbers. Of note, however, if these persons did not respond because they were away from their landline telephones, their inclusion would have resulted in even stronger associations. Among controls, refusal to participate was uncommon (12.7%); vacant residences were the main cause of nonresponse. Although interviews occurred on weekends, the limited attempts made to locate absent residents could have resulted in enrollment of controls who were more likely to practice social distancing. If so, this factor could have resulted in an overreport of the true effect. However, other reasons could explain the high vacancy rate, such as residents visiting vacation homes or relatives; residents, especially in apartments or other housing with restricted access, not responding to strangers; and residences being temporarily vacant. Our adjustment for age, sex, and other covariates could have at least partially controlled for these differential responses.

Second, some exposure misclassification was possible, because questions about mask use and social distancing were unvalidated and limited in detail, having been taken from the community serology survey providing the controls. As such, we were unable to address differences in protection when indoors, outdoors, or indoors in specific settings.

Third, full adjustment for pandemic moment in analyses of mask use was not possible, because controls with data on mask use were all interviewed in a period shortly after case-patients began experiencing symptoms. However, given that this period was short and followed mandated mask use, a temporal trend in mask use would probably have been small and thus have had little effect on our estimates.

Fourth, controls could include persons who had received a misdiagnosis of false-negative. However, given low seroprevalence and our test’s 86.4% sensitivity and 99.6% specificity (L.C. Pellanda, unpub. data), we estimate that misdiagnosis would likely have occurred in only 1 control.

Fifth, as the serology survey did not include occupation, we could not exclude healthcare workers among controls. Because healthcare workers would likely adhere to greater social distancing and mask use, their inclusion among controls could have falsely strengthened our findings. However, only ≈5% of the workforce in Brazil are healthcare workers (*25*), so we do not believe that their inclusion produced an appreciable error. In addition, errors because of lack of control for unmeasured confounding (e.g., from other occupational or residual socioeconomic differences or from recent travel) are always possible.

Sixth, a specific finding—lesser protection of those who reported being practically isolated from everyone and those who reported staying at home all the time (Table 3, model 3)—could weaken confidence in our social distancing results. However, as suggested by the additional analysis removing proxy responses (model 4), the lack of a graded dose-response in the model 3 associations could have been because of the greater risk level of case-patients who reported they were isolated but actually resided in assisted living.

Finally, application of our findings to settings with circulating virus variants or to persons who have received vaccination can only be speculated. In the case of circulating variants, risk for infection among those distancing and using masks will probably be greater, but risk among those not distancing or not using masks will also be greater. In the case of vaccination, however, risk for infection would be lower for all. We know of no a priori reason, however, to presume that the relative protection of distancing and mask use in these settings would be either lesser or greater than we report.

The primary strength of our report is that, as a population-based study, it avoids the risk for selection bias typical of less representative designs. It is sufficiently large to permit precise confidence intervals for our estimates of the benefit of protective measures. Furthermore, because our cases were detected at a time when testing was limited to symptomatic persons seeking care, the protection we found was against becoming a clinically relevant case. Finally, and perhaps most vital, our findings are based on individual-level analyses and thus permit estimation of percent reduction of risk, a direct and simple way to communicate the magnitude of individual protection afforded by these simple public health measures.

Given the hurdles faced in vaccine production, distribution, and acceptance, and the increasing emergence of virus variants, mass vaccination is unlikely to suffice to control the pandemic in the near future in many parts of the world. During this period and continuing into the future phase of maintaining viral control, simple public health measures, principally social distancing and mask use, will remain crucial options to minimize viral spread.

In conclusion, we found that social distancing and mask use while away from home provided major protection against symptomatic SARS-CoV-2 infection. Our easily grasped and generalizable estimates of protection against transmission lend support to previous, frequently less direct, assessments. Our findings support the contention that greater use of simple public health measures in the community provides major protection against symptomatic infection.
